# Under-recognition of medically unexplained symptom conditions among US Veterans with Gulf War Illness

**DOI:** 10.1371/journal.pone.0259341

**Published:** 2021-12-07

**Authors:** Naomi S. Kane, Nicole Anastasides, David R. Litke, Drew A. Helmer, Stephen C. Hunt, Karen S. Quigley, Wilfred R. Pigeon, Lisa M. McAndrew

**Affiliations:** 1 VA New Jersey Health Care System, War Related Illness and Injury Study Center, East Orange, NJ, United States of America; 2 Department of Rehabilitation Medicine, New York University Grossman School of Medicine, New York, NY, United States of America; 3 Michael DeBakey VA Medical Center, Center for Innovations in Quality, Effectiveness, and Safety (IQuESt), Houston, TX, United States of America; 4 VA Puget Sound Health Care System, Seattle, WS, United States of America; 5 Department of Medicine, University of Washington, Seattle, WS, United States of America; 6 VA Bedford Healthcare System, Center for Health Organization & Implementation Research (CHOIR), Bedford, MA, United States of America; 7 Department of Psychology, Northeastern University, Boston, MA, United States of America; 8 Finger Lakes Healthcare System/VISN 2 Center of Excellence for Suicide Prevention, Canandaigua, NY, United States of America; 9 Psychiatry Department, University of Rochester Medical Center, Rochester, NY, United States of America; 10 Department of Educational and Counseling Psychology, University at Albany, Albany, NY, United States of America; University of California, San Francisco, UNITED STATES

## Abstract

**Objective:**

Conditions defined by persistent “medically unexplained” physical symptoms and syndromes (MUS) are common and disabling. Veterans from the Gulf War (deployed 1990–1991) have notably high prevalence and disability from MUS conditions. Individuals with MUS report that providers do not recognize their MUS conditions. Our goal was to determine if Veterans with MUS receive an ICD-10 diagnosis for a MUS condition or receive disability benefits available to them for these conditions.

**Methods:**

A chart review was conducted with US Veterans who met case criteria for Gulf War Illness, a complex MUS condition (*N* = 204, *M* = 53 years-old, *SD* = 7). Three coders independently reviewed Veteran’s medical records for MUS condition diagnosis or service-connection along with comorbid mental and physical health conditions. Service-connection refers to US Veterans Affairs disability benefits eligibility for conditions or injuries experienced during or exacerbated by military service.

**Results:**

Twenty-nine percent had a diagnosis of a MUS condition in their medical record, the most common were irritable colon/irritable bowel syndrome (16%) and fibromyalgia (11%). Slightly more Veterans were service-connected for a MUS condition (38%) as compared to diagnosed. There were high rates of diagnoses and service-connection for mental health (diagnoses 76% and service-connection 74%), musculoskeletal (diagnoses 86%, service-connection 79%), and illness-related conditions (diagnoses 98%, service-connection 49%).

**Conclusion:**

Given that all participants were Gulf War Veterans who met criteria for a MUS condition, our results suggest that MUS conditions in Gulf War Veterans are under-recognized with regard to clinical diagnosis and service-connected disability. Veterans were more likely to be diagnosed and service-connected for musculoskeletal-related and mental health conditions than MUS conditions. Providers may need education and training to facilitate diagnosis of and service-connection for MUS conditions. We believe that greater acknowledgement and validation of MUS conditions would increase patient engagement with healthcare as well as provider and patient satisfaction with care.

## Introduction

Conditions characterized by persistent ‘medically unexplained’ physical symptoms (MUS) are prevalent and disabling, impacting an estimated 40–50% of patients in primary care [1]. They are particularly relevant for Veterans who deployed to the Persian Gulf region, where 30% or more returned with multiple chronic and disabling symptoms (e.g., fatigue, chronic pain) [2–4]. MUS is an umbrella term for symptom-based conditions (also referred to as functional somatic disorders and persistent physical symptoms, among others [5,6]). There are several delimitated MUS conditions, such as fibromyalgia, irritable bowel syndrome and Gulf War Illness. Existing case definitions for these conditions significantly overlap and patients typically meet criteria for more than one case definition [7,8]. While historically, the cause has been unknown, there is growing evidence that MUS conditions are likely explained by multiple determinants including immunological and central nervous system abnormalities [9–11].

Despite increased understanding of the multiple factors that contribute to MUS, there are not yet established biomarkers that can definitively identify MUS conditions which creates diagnostic challenges for providers. Extant research finds providers have over-diagnosed patients who do not meet the case definition for a MUS condition, as having a MUS condition [12–14]. It is also likely that providers underdiagnosed MUS conditions or misdiagnose MUS conditions as a mental health or a more familiar medical condition [15–18].

There are reasons to suspect that providers do not recognize patients as having MUS conditions. Patients with MUS conditions have twice the amount of healthcare utilization compared to patients with better understood conditions [19], much of this care is thought to be the result of providers not recognizing patients as having a MUS condition and instead trying identify potential causes for the symptoms (e.g., unidentified multiple sclerosis, cancer) [19,20]. When the search for the cause is unsuccessful, patients often describe their provider as concluding that their condition is “all in their head” or a “mental health condition” [21]. Providers, in turn, report that they were untrained to recognize or treat MUS conditions [22]. Patients and providers both describe negative experiences due to an inability to develop a common understanding of MUS conditions [23].

Initial research supports the hypothesis that MUS conditions are underdiagnosed. For example, a study of 529 initial patient encounters found that providers’ provisional diagnoses missed MUS conditions over 50% of the time [24]. Another study found that among individuals who meet the case definition for a MUS condition, only 14% reported having previously been diagnosed with a MUS condition [25]. To our knowledge there is only one study that examined rates of ICD-10 diagnosis for patients with a MUS condition. This study found only 50% of patients with fibromyalgia seen in one rheumatology clinic received an ICD-10 diagnosis for fibromyalgia [26].

Gulf War Veterans should, presumably, be recognized as having MUS conditions as US law presumes MUS is a consequence of deployment to the Gulf [27]. Gulf War Veterans with MUS are eligible for no-cost healthcare and monthly disability benefits, termed “service-connection” [28]. Any MUS condition including fibromyalgia, irritable bowel syndrome, and chronic fatigue syndrome is eligible for service-connection. The MUS condition of Gulf War Veterans is typically termed Gulf War Illness, or Chronic Multisymptom Illness, and a case definition has been created which overlaps with other case definitions for MUS conditions [2,29,30]. Despite recognition from Congress, Veterans often report being frustrated that their MUS condition is not acknowledged by their providers and receiving limited disability benefits for their MUS condition [31–33]. Further, the military exposures that lead to MUS are under-recognize by providers [34].

Our objective was to review the medical records of Veterans who meet the case definition for Gulf War Illness to determine the prevalence of diagnosis or service-connection for any MUS condition (i.e., fibromyalgia, irritable colon/irritable bowel syndrome, and chronic fatigue syndrome) and the agreement between diagnosis and service-connection for MUS conditions. While Gulf War Illness is not recognized in the ICD-10 classification, MUS conditions that are in the ICD-10 have considerable overlap with Gulf War Illness and are considered by Congress to be caused by deployment to the Gulf [28]. We contrast the rates of diagnosis and service-connection for MUS conditions to rates of diagnosis and service-connection for comorbid mental or physical health conditions. Low rates of clinical diagnosis for MUS conditions among this cohort may indicate that recognition is a barrier to receiving care and service-connected benefits.

## Methods

### Participants

Participants were enrolled in a larger (*N* = 268) interventional study examining the effectiveness of Problem-Solving Treatment to reduce disability for Veterans with Gulf War Illness [31,35–37]. To be eligible for the study, Veterans had to 1) deploy to Operation Desert Storm or Operation Desert Shield between August 1990 and November 1991, 2) meet the case definition for Gulf War Illness [2], and 3) report disability on the World Health Organization Disability Assessment Schedule (WHODAS 2.0) [38]. Gulf War Illness was defined using the Kansas criteria for Gulf War Illness which includes symptoms in at least three out of the following six domains: fatigue, pain, neurological/cognitive/mood, skin, gastrointestinal, and respiratory [2]. In the present analyses, we excluded Veterans who were lost to follow up (*n* = 29), died during the course of the study (*n =* 1), were not enrolled in VA care (*n* = 22) or did not have any service-connected conditions (*n* = 12). The final sample described below included 204 Veterans.

### Setting and procedures

Study personnel screened Veterans to determine if they met the case definition for Gulf War Illness. Veterans were screened and enrolled at one of three Veterans Affairs Medical Centers (VAMCs) in the northeastern United States, each of which provided IRB approval. Veterans could be receiving care at any VAMC or not receiving VA care. The Veteran provided written consent for the research study. The second author (NA) extracted problem list diagnoses and service-connection conditions from the VA’s national electronic medical record system.

**Fig 1:**
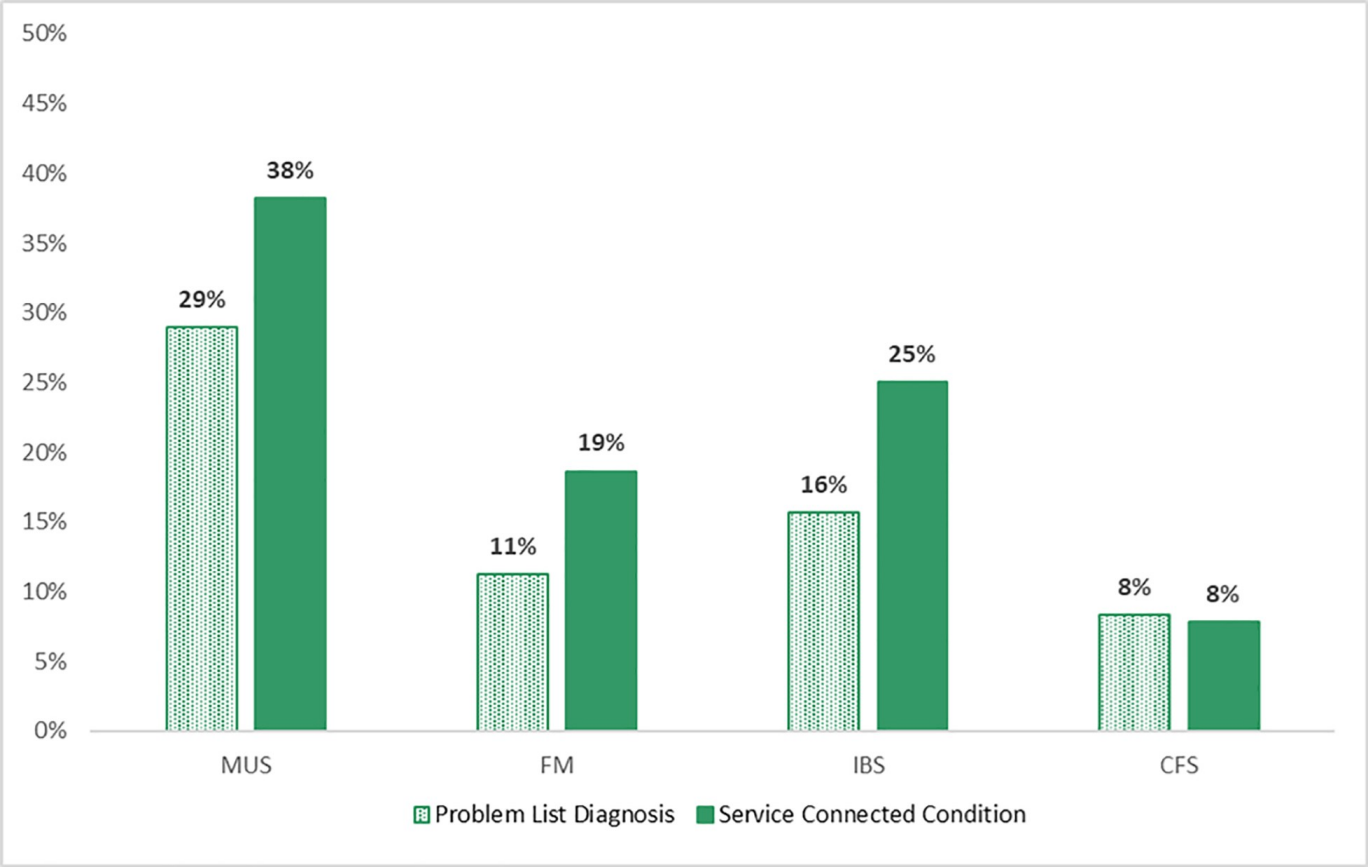
Problem list diagnoses and service-connection for medically unexplained symptoms/conditions. MUS = medically unexplained symptoms/conditions, FM = fibromyalgia, IBS = irritable bowel syndrome or irritable colon, CFS = chronic fatigue syndrome.

The first and second authors created a coding scheme by which problem list diagnoses and service-connected conditions were organized **(Fig 1**). Conditions were organized as follows: A) MUS conditions that are presumed service-connected by the VA including chronic fatigue, fibromyalgia, irritable colon/irritable bowel syndrome; B) migraine; C) mental health disorders including but not limited to depression, post-traumatic stress disorder, and adjustment disorders; D) musculoskeletal and mechanical related injuries including muscle inflammation, prosthesis, traumatic brain disease; and E) illness-related conditions, or medical conditions that are generally considered to be medically explained including cancer, respiratory conditions, and skin or vascular abnormalities. Migraine was included as a separate category due to its high prevalence among this population [39]. Raters consulted with two physicians (DH, SH), who have direct clinical and research experience with Gulf War Veterans to create the coding list. Three raters independently reviewed diagnoses and service-connected conditions. Raters then met to resolve discrepancies and develop agreement for categorizing new diagnoses as they arose.

Chi-square tests were used to assess differences in the proportion of the sample with a diagnosis and the proportion of the sample who were service-connected [40]. We also assessed Cohen’s kappa (κ) [41] which is a measure of concordance. That is, it is a measure of if Veterans received or did not receive both a diagnosis and service-connection, as compared to only receiving a diagnosis or service-connection. We used SPSS version 25 and reported *p*-values.

## Results

This sample of Gulf War Veterans was predominantly male, White, and middle aged **(Table 1)**, which is generally representative of this population [42].

**Table 1:** Descriptive statistics for the demographic and health characteristics for the *N* = 204 sample.

Variables		Range
Age, mean (SD), *n* = 202	52.8 (7.1)	42–79
Ethnicity, *n* (%), *n* = 203		
Non-Hispanic White	*174* (85.7%)	
Race, *n* (%), *n* = 202		
White	*145* (71.8%)	
Black	*43* (21.3%)	
Hispanic	*11* (5.5%)	
American Indian	*10* (5%)	
Asian	*3 (*1.5%)	
Native Hawaiian	*1* (0.5%)	
More than one race	*9* (4.5%)	
Female, *n* (%), *n* = 202	*28* (13.5%*)*	
Education, *n* (%), *n* = 202		
Some high school	*1* (0.5%)	
High school	*23* (11.1%)	
Some college	*58* (28%)	
College graduate	*62* (30%)	
Trade, technical, or vocational training	*13* (6.3%)	
Some graduate school	*11* (5.3%)	
Graduate degree	*33* (15.9%)	
Some college and trade school	*5* (2.5%)	
College graduate and trade school	*3* (1.5%)	
Service-connection percentage, mean (SD), *n =* 204	77.4 (22.4)	10–100

As shown in Fig 1, 28.9% of the sample was diagnosed with a MUS condition including, 15.7% diagnosed with irritable colon/irritable bowel syndrome, 11.3% diagnosed with fibromyalgia, and 8.3% diagnosed with chronic fatigue syndrome. The proportion service-connected was slightly higher than diagnosed, with a total of 38.2% of the sample service-connected for a MUS condition (*χ*^2^(1) = 3.97, *p =* .046) including irritable bowel syndrome/colon (25%; *χ*^2^(1) = 5.46, *p =* .02), fibromyalgia (18.6%; *χ*^2^(1) = 4.34, *p =* .04), and chronic fatigue syndrome (7.8%; *χ*^2^(1) = .04, *p =* .86). The concordance (Cohen’s kappa) was moderate for MUS conditions (κ = .60, *p* < .001). That is, individuals who were diagnosed with a MUS condition were also likely to be service-connected for a MUS condition. The concordance (Cohen’s kappa) was also moderate for specific MUS conditions including fibromyalgia (κ = .52, *p* < .001), irritable bowel syndrome/colon (κ = .51, *p* < .001), and chronic fatigue syndrome (κ = .57, *p* < .001).

**Fig 2:**
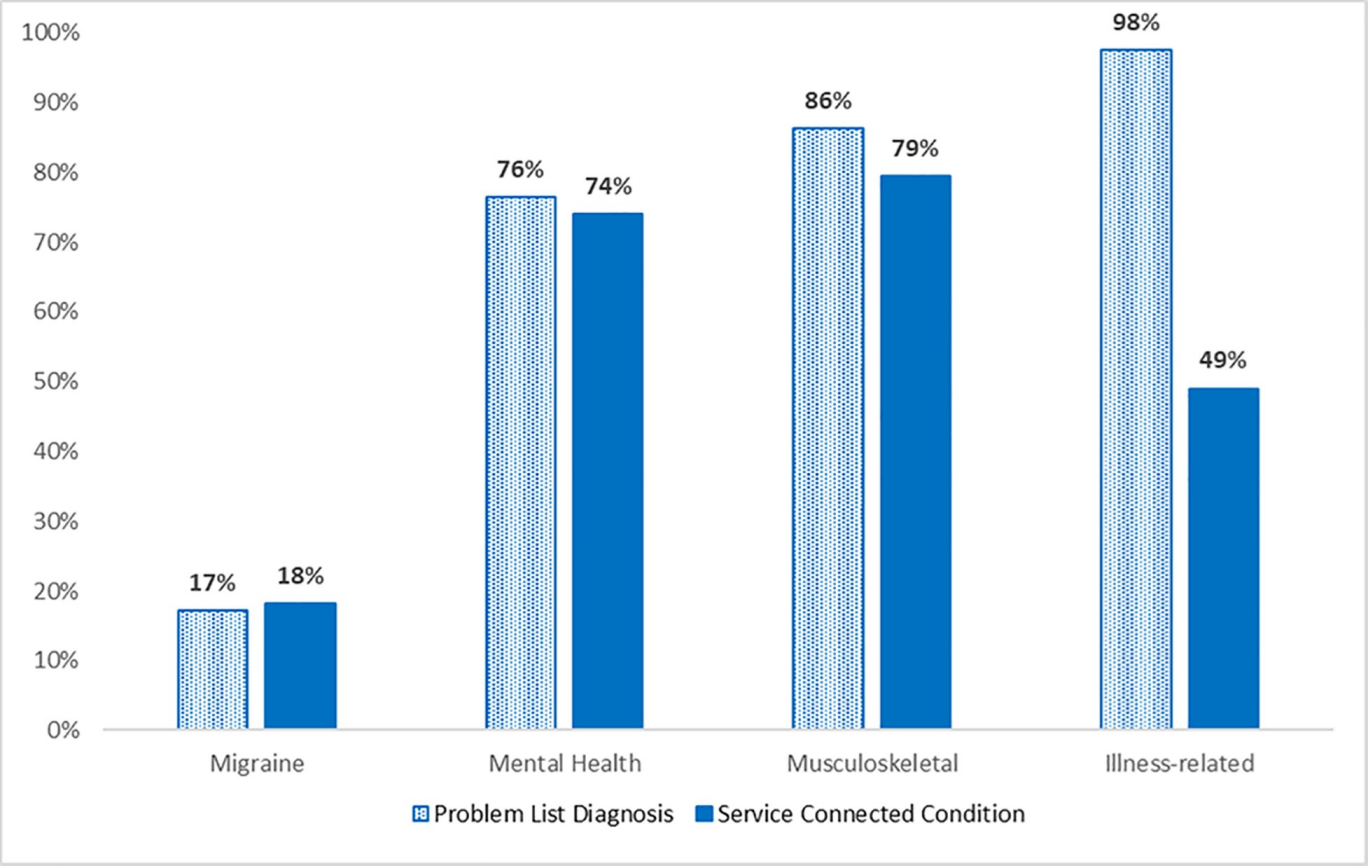
Problem list diagnoses and service-connection for migraine, mental health conditions, musculoskeletal/mechanical injuries, and illness related conditions.

As shown in Fig 2, slightly less than one-fifth of the sample had a diagnosis of a migraine condition (17.2%) while most received a diagnosis for a mental health (76.5%), musculoskeletal (86.3%) and illness-related condition (97.5%). The proportion of the Veterans who were service-connected was similar to the proportion who received a clinical diagnosis for migraine (18.1%; *χ*^2^(1) = .07, *p =* .80), mental health (74.0%; *χ*^2^(1) = .33, *p =* .57), and musculoskeletal conditions (79.4%; *χ*^2^(1) = 3.38, *p =* .07), but dissimilar for illness-related conditions (49%; *χ*^2^(1) = 122.70, *p* < .001). The concordance (Cohen’s kappa) was moderate for migraine (κ = .43, *p* < .001), and mental health diagnoses (κ = .41, *p* < .001), with low concordance for musculoskeletal conditions (κ = .18, *p* = .01). The concordance for illness-related diagnoses was very low (κ = .03 *p* = .19). That is, individuals diagnosed with migraine and mental health conditions were also likely to be service-connected for these conditions, while those diagnosed with an illness-related condition were not likely to be service-connected for these conditions.

## Discussion

We found that in a sample of Gulf War Veterans who our study team determined met criteria for a MUS condition, only 29% had a diagnosis in their medical record for any MUS condition. The most common MUS diagnosis was irritable colon/irritable bowel syndrome (16%) followed by fibromyalgia (11%). This was in stark contrast to higher rates of diagnoses for mental health (76%), musculoskeletal (86%), and illness-related conditions (98%). Veterans were slightly more likely to be receiving disability benefits, termed service-connection in the US Department of Veterans Affairs, for a MUS condition (38%) than diagnosed with a MUS condition. To our knowledge, there are no published reports of rates of diagnosis or service-connection for MUS conditions among Gulf War Veterans.

Together these results suggest that Veterans of this conflict infrequently receive a diagnosis for MUS conditions. This is important given patients’ frustration about receiving care for these difficult to diagnose conditions [32,43]. One reason may be that providers are reluctant to diagnose a MUS condition due to questions about whether this is the best diagnosis [23] and concern that they are missing a medically explained condition. Providers may also lack knowledge about MUS case definitions, such as the criteria for fibromyalgia [44]. Providers report little training in MUS and not feeling expertise in treating MUS conditions [45]. They also may be reluctant to diagnose patients with a MUS condition, as the patient may perceive it as stigmatizing.

Another reason for the low rates of diagnosis for MUS conditions may be confusion and controversy over terminology. Historically, MUS was termed somatization, a psychological condition. This fell out of favor and was replaced by medically unexplained symptoms and conditions. Growing recognition that the cause of MUS conditions is multi-determined [11], has led to calls for the use of terms such as “persistent physical symptoms” and “functional somatic syndromes,” among others [5,6,46,47]. Case definitions for specific types of MUS have also evolved, such as the criteria to diagnose fibromyalgia [44]. The medical community’s evolving understanding of, and definitions for, MUS likely contributes to low diagnoses for these conditions.

Differing opinions from providers about the etiology of MUS conditions may also contribute to underdiagnosis. For example, it has been shown that mental health providers are more likely to attribute Gulf War Illness to physical exposure, while internal medicine clinicians are more likely to attribute Gulf War Illness to stress or a psychological condition [15]. Provider attribution of MUS conditions to other more commonly diagnosed conditions is important given that patients with MUS conditions have many comorbid conditions [48]. An estimated 50%-60% of patients with MUS conditions [49], including combat Veterans [4], have a mental health condition. While studies confirm that MUS and mental health conditions are distinct, patients with MUS conditions often feel providers dismiss, and misattribute their MUS condition to a mental health condition [21]. Individuals in the current study had a higher prevalence of mental health diagnosis and/or musculoskeletal diagnosis than a diagnosis for a MUS condition. It remains unclear whether providers were correctly diagnosing Veterans with comorbid conditions or if they were simply attributing the MUS condition to a mental health or other condition.

The reasons for underdiagnosis of Veterans with MUS conditions may be particularly complex. The Veterans Health Administration and the Veterans Benefits Administration are inconsistent in their choice of terminology, with medically unexplained symptoms being used by the Veterans Benefits Administration and Gulf War Illness or Chronic Multisymptom Illness being more commonly used in the Veterans Health Administration. The inconsistent terminology likely complicates diagnosis. Further, there is no ICD-10 code for Gulf War Illness. While the case definition for Gulf War Illness significantly overlaps with other MUS condition case definitions (e.g., fibromyalgia), it is possible that providers felt Gulf War Illness was the correct diagnosis so did not enter a ICD-10 diagnosis for a MUS condition. We suspect, however, that as opposed to providers having a nuanced understanding of different MUS case definitions, they simply did not recognize Veterans as having any MUS condition. In support of this, most providers of Veterans report not having adequate knowledge of Gulf War Illness or MUS [50].

In addition to underdiagnosis, most of the Veterans in our sample were not service-connected for a MUS condition despite meeting a case definition for a MUS condition (Gulf War Illness) and endorsing disability that would appear to make them eligible. Legislative intent is to provide accessible healthcare and monetary benefits for Veterans who have physical and mental health concerns resulting from their deployment [28,51]. There is evidence that this intent is successful. For example, Vietnam War Veterans who are service connected show higher rates of VA healthcare utilization than those who are not service connected [52]. However, if the system, i.e., Veterans Benefits Administration, and individual-level providers are not recognizing MUS conditions, Veterans with these conditions cannot receive the no cost care they are entitled to for these conditions that are caused or exacerbated because of service. Future investigation should examine if low rates of service-connection for MUS conditions are due to Veterans not being aware of potential service-connection, not applying, the presence of barriers in the MUS claim evaluation and adjudication process, or if Veterans are not being clinically assessed and informed of these conditions and their potential relation to military service.

We found that Veterans who were service connected for a MUS condition were more likely to also have a documented clinical diagnosis of a MUS condition. This is perhaps due to greater access to healthcare, providing more opportunities for a diagnosis for a MUS condition. It may also be possible that providers consider a MUS condition diagnosis after seeing a MUS-related service-connection. However, because this study was cross-sectional, we cannot know if service-connection facilitated clinical diagnosis or vice versa.

This study suggests that Veterans with Gulf War Illness are underdiagnosed with MUS conditions and few are service-connected. A limitation is that our results cannot determine if this underdiagnosis impacts care. Clinical practice guidelines for MUS conditions recommend non-pharmacological treatments to improve functioning and quality of life and limiting unnecessary assessment. It is not known if giving providers support and tools to allow them to correctly identify MUS conditions would facilitate better treatment. Theoretically, a diagnosis of a MUS condition is not needed to follow clinical practice guidelines and it is likely that some of the sample who did receive a MUS condition diagnosis were receiving guideline congruent care. However, in general, individuals with MUS conditions do not receive guideline congruent care. Instead, they often receive excessive healthcare [19], overly focused on diagnostic tests to determine the cause of the MUS [20]. Studies have found that individuals prefer explicit diagnoses such as chronic fatigue syndrome over the uncertainty of no diagnosis [53], and that they find acknowledgement and validation to be the most helpful communication from their provider [31]. How diagnosis for a MUS condition would influence Veterans’ care is unknown.

While not knowing the generalizability of the results is a limitation, finding underdiagnosis among a population presumed by the US Congress to be at high risk for MUS conditions suggests that under-diagnosis of MUS conditions may be a generalized problem for all patients with MUS conditions. Future studies should examine rates of diagnoses of MUS conditions among civilians. Another limitation is that while we examined rates of MUS condition diagnosis among a sample who met a case definition for a MUS condition, it is possible that some of the Veterans may have an underlying condition that accounted for their MUS that had not yet been diagnosed or recognized by the study team. We also were unable to access medical records for individuals seeking care outside of the VA. Some of the Veterans in this study may also have been service-connected for a MUS condition and not have had this yet been entered into the medical record, although this information is updated regularly. Future studies should explore the relationship between clinical diagnosis for MUS conditions, service-connection for MUS conditions, and satisfaction/quality of care for MUS conditions with emphasis on qualitative methods to determine provider and patient attitudes and beliefs about MUS condition diagnosis.

In conclusion, this review of the medical records of Veterans with a MUS condition suggests that MUS conditions are under recognized in clinical and disability settings. Providers may need support to facilitate diagnosis of MUS conditions. Greater acknowledgement and validation of MUS conditions may help to increase patient engagement with healthcare, provider satisfaction and ultimately improve outcomes for individuals living with theses challenging health condition.
